# San Antonio refugees: Their demographics, healthcare profiles, and how to better serve them

**DOI:** 10.1371/journal.pone.0211930

**Published:** 2019-02-19

**Authors:** Fadi W. Adel, Eden Bernstein, Michael Tcheyan, Shane Ali, Heidi Worabo, Moshtagh Farokhi, Andrew E. Muck

**Affiliations:** 1 Department of Internal Medicine, Mayo Clinic, Rochester, Minnesota, United States of America; 2 Department of Internal Medicine, Cleveland Clinic, Cleveland, Ohio, United States of America; 3 The San Antonio Refugee Health Clinic, The Student-Faculty Collaborative Practices Program, The Center for Medical Humanities and Ethics, The Long School of Medicine, The University of Texas Health San Antonio, San Antonio, Texas, United States of America; University of Nebraska Medical Center, UNITED STATES

## Abstract

**Objective:**

The recent refugee crisis has resulted in the largest burden of displacement in history, with the US being the top resettlement country since 1975. Texas welcomed the second most US-bound refugees in 2016, with a large percentage arriving in San Antonio. Yet, the composition of the San Antonio refugees has not been described and their healthcare needs remain ill-defined. Through this study, we aim at elucidating their demographics and healthcare profiles, with the goal of devising recommendations to help guide refugee program development and guide other refugee resettlement programs.

**Methods:**

Data from 731 charts belonging to 448 patients at the San Antonio Refugee Health Clinic (SARHC) were extracted and analyzed. Data included age, gender, country of origin, first language, interpretation need, health insurance status, medical history, vital signs, diagnoses, and prescribed medications.

**Results:**

Women constituted the majority of patients (n = 267; 56.4%), and the median age of all patients was 39 (Q1:26, Q3:52). Nepali-speaking Bhutanese patients were the most represented group (n = 107, 43.1%), followed by Iraqi (n = 35, 14.1%), Burmese (n = 30, 12.1%), and Iranian (n = 19, 7.7%) refugees. Of those who responded, 200 (86.6%) did not have any form of health insurance. Additionally, 262 (50.9%) had a body-mass index (BMI) in the overweight or obese range. Further, 61.4% (n = 337) had blood pressures in the hypertensive range, while 9.3% (n = 51) had an elevated blood pressure. On average, each patient had 1.9 complaints, with abdominal pain, headaches, and cough being the predominant complaints. Allergic rhinitis, viral upper respiratory infections, and elevated blood pressure were the most common diagnoses. However, the list of common diagnoses differed per country of origin.

**Conclusion:**

The SARHC demographics were different from those of other Texas refugees. The rate of the uninsured and the burden of non-communicable diseases were high. Furthermore, each refugee subgroup had a different set of common problems. These findings reveal important considerations for refugee healthcare providers and the unique approach that may be required for different communities.

## Introduction

Persecution, war, and violence have driven people from their home countries in search of a safer refuge, leading to the largest burden of displacement witnessed by the United Nations High Commissioner for Refugees (UNHCR) [1]. There are more than 21.3 million refugees in the world, less than 1% of whom have the opportunity of being resettled. More than 3 million refugees have resettled in their new homes in the US since 1975, making the US the top resettlement country in the world. In 2016, Texas received the second highest number of US-bound refugees (n = 7,802) after California (n = 7,909) [2], as the US welcomed 84,994 refugees [3]. Specifically, Bexar County, which includes San Antonio, hosted more than 1,000 refugees, 12% of which received no health screening whatsoever. TB, Syphilis, HIV, and elevated lead levels were amongst the common problems of the Texas refugee population [4].

While resettlement is stressful due to language and cultural barriers [5], many refugees cite healthcare as their most vital issue [6]. Additionally, the unstable living conditions of refugees predispose them to a multitude of diseases, such as tuberculosis and gastrointestinal infections [7–9], mental health issues [10], malnutrition [11] and hypertension [12].

Of note, under current Texas guidelines, most refugees lose Medicaid coverage after 8 months of resettlement [13], rendering a large portion of this population without any health insurance.

Given the aforementioned data and the complex barriers to healthcare access in the US, newly-resettled refugees face significant difficulties [14]. Moreover, the withdrawal of Texas from the federal Refugee Resettlement Program complicated matters even more and left the fate of resettlement services up to the local non-profit organizations. This can translate to discontinuity of services, at least temporarily [15]. With these gaps in healthcare coverage for the refugee population, the San Antonio Refugee Health Clinic was established in 2012 to act as the safety net for local refugees. It is a Student-Faculty Collaborative Practice (SFCP) where medical, dental, nursing, and physician assistant students and faculty at the University of Texas Health San Antonio serve the mostly uninsured and underserved refugee population of San Antonio, Texas. The clinic utilizes the site of a local church in San Antonio, Texas on a weekly basis to serve the refugees who live in the surrounding areas.

The goal of our study was to gain a better understanding of the San Antonio refugee population by inspecting a sample of refugees’ profiles who are also patients at the SARHC. This retrospective chart review (RCR) was undertaken to delineate countries of origin, spoken languages, common symptomatology, social histories, insurance status, common diagnoses, labs ordered, prescribed medications, and the prevalence of certain chronic diseases. The better understanding of this unique community will allow us to better serve the unique needs of this population and will hopefully provide some background for planning of similar clinics in other locations.

## Materials and methods

Prior to the initiation of chart reviews, an approval for exempt status by the Institutional Board Review (IRB) at The University of Texas Health San Antonio was obtained. Electronic patient charts at the clinic since its inception in February, 2012 through May, 2016 were reviewed. Briefly during this time period, paper charts were used, but those were not included in this study. The IRB waived the requirement for informed consent. The collected data were, however, anonymized at the time of collection and included in a database which contained the month and year of the visit, age at first visit, country of origin, first language, interpreter utilization, insurance status, sex, height, weight, body mass index (BMI), vitals on admission (temperature, respiratory rate, heart rate, blood pressure (BP), oxygen saturation, blood glucose when indicated), chief complaint, diagnoses, onsite testing, offsite testing, medications used by the patient, prescribed medications, past medical history findings, past surgical history findings, specialty referrals, health insurance status, tobacco and alcohol use, and reasons for follow-up at the clinic.

The collected data were then analyzed using Microsoft Excel. The patients were stratified into 10-year age groups (1–10 year olds, 11–20 year olds, etc), and divided based on their country of origin, first languages, gender, and BMI distribution.

Further, vitals on admission were inspected and the following cutoffs were used: a temperature reading above 100.4 ⁰Fahrenheit (38 ⁰Celsius) constituted fever; tachycardia was defined as a heart rate > 100 beats per minute (bpm) and bradycardia as a heart rate < 60 bpm; tachypnea was defined as a respiratory rate > 20 breaths per minute; and the 2017 American College of Cardiology /American Heart Association (ACC/AHA) guidelines on BP were adopted to define abnormal blood pressure [16].

The chief complaints (CC) were grouped into the following categories based on a classification devised by the authors: gastrointestinal complaints (abdominal pain, constipation, heart burn, diarrhea, hematochezia, dysphagia, nausea, vomiting, flatulence, indigestion); musculoskeletal pain (lower and upper extremity pain, arthralgia, shoulder pain, bone pain); upper respiratory disease symptoms (cough, sore throat, nasal congestion); dermatological complaints (rash, itching, “lumps”, blisters, hair loss, eyelid bumps, depigmentation, dry skin, ulcers); constitutional symptoms (generalized pain, fever, shortness of breath, chills, sweating, weight loss, weight gain, lymphadenopathy); headache; dental complaints (toothache, dental check-up, oral pain, oral lesion); follow-up; cardiovascular concerns (hypertension, chest pain, lower extremity edema, high glucose, high cholesterol); medication refill; genitourinary and obstetric complaints (dysuria, dysmenorrhea, polyuria, flank pain, breast pain, vaginal discharge, pregnancy, genital pain, vaginal itching, contraception, dyspareunia, genital warts, hematuria, nocturia); back pain; neurological complaints (neck pain, dizziness, numbness, weakness, burning feet, difficulty walking, dysgeusia); vision problems (itchy eyes, vision loss, eye pain, cataract, droopy eyelids); general check-up (sports physical, medicine recommendations, BP check, job clearance forms); psychiatric problems (depression, bipolar disorder, memory issues, insomnia), and other.

The running diagnoses were compiled and the most common ones were presented. Further, the diagnoses of the patients from the five most represented countries of origin were presented and compared with one another. The on-site testing (urine Beta-human chorionic gonadotropin (BhCG), dipstick urinalysis, glucometer, and rapid strep throat kit) and off-site testing were also compiled and presented (S1 Table). The most commonly prescribed medications and the patients’ social histories were also extracted.

## Results

### Demographics

Overall, 731 charts were reviewed and included in this study, pertaining to 448 patients, some of whom had multiple visits to the SARHC. The mode of the number of visits was a single visit (n = 448, 61.4%), with 16.8% (n = 123) being second visits, and one patient having up to 17 visits.

**Fig 1:**
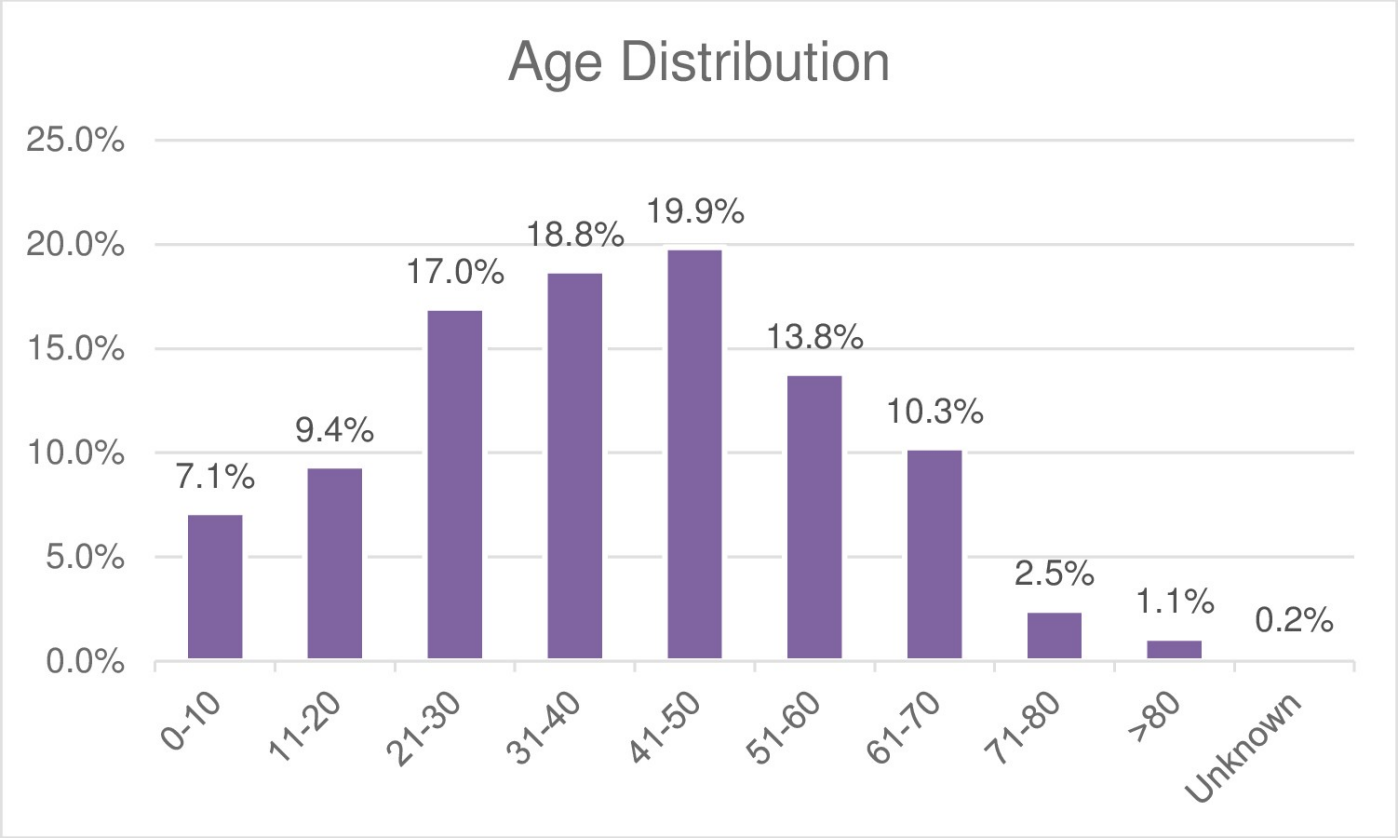
The age distribution of the SARHC patients. The distribution is semi-normal, with more patients falling in the 41–50 year-old range (n = 89; 19.9%). Twenty-six countries were represented, with Nepali-speaking Bhutanese refugees comprising the biggest cohort (n = 107, 43.1%), followed by Iraqi (n = 35, 14.1%), Burmese (n = 30, 12.1%) and Iranian (n = 19, 7.7%) refugees (Table 1).

One hundred and seventy-nine (40%) were male, and 267 (56.4%) were female. The median age at the first visit was 39 (min:2 months; max: 90) (Fig 1).

**Table 1:** The distribution of SARHC patients by their country of origin.

Country of origin	Number of patients (%)
Bhutan	107 (43.1)
Iraq	35 (14)
Burma	30 (12.1)
Iran	19 (7.7)
Thailand	11 (4.4)
Central African Republic	8 (3.2)
Cameroon	4 (1.6)
Ethiopia, Chad, Congo, Somalia, Sudan	3 each (1.2 each)
Afghanistan, Bangladesh, Lebanon, Morocco, Syria	2 each (0.8 each)
Colombia, Cuba, Eastern African country*, India, Jordan, Liberia, Rwanda, South Africa, Sri Lanka	1 each (0.4 each)

* Respondent simply stated “East Africa”

The languages spoken by patients followed the country of origin’s distribution, with 104 refugees speaking Nepali (43%), 41 speaking Arabic (16.9%), 21 speaking Burmese (8.7%), 16 speaking Farsi (n = 6.6%), and 15 speaking French (6.2%). For a full listing of languages spoken, see Table 2.

**Table 2:** Distribution of languages spoken by patients at the SARHC.

First Language	Frequency (%)
Nepali	104 (43.0)
Arabic	41 (16.9)
Burmese	21 (8.7)
Farsi	16 (6.6)
French	15 (6.2)
Karen	13 (5.4)
Tigrigna	10 (4.1)
Swahili	4 (1.7)
Somali	3 (1.2)
Bengali, Oromo, Spanish, Thai	2 each (0.8 each)
Amharic, Aramaic, Burundian, English, Hindi, Tamil	1 each (0.4 each)

### Language interpretation

While not all patients indicated whether or not they needed interpretation, of those who responded (n = 201), 49.8% needed an interpreter and 5.5% preferred one, with 44.8% not needing one.

### Insurance and primary care providers (PCP)

Of those who responded (n = 231), 86.6% (n = 200) did not have any form of insurance. Only 12 patients had Medicaid (5.2%), and 12 others had a Bexar County-specific healthcare subsidy program. Additionally, only 12.3% (n = 30) patients had a PCP, almost corresponding to the number of patients who had insurance.

### Vitals during clinic visit

**Fig 2:**
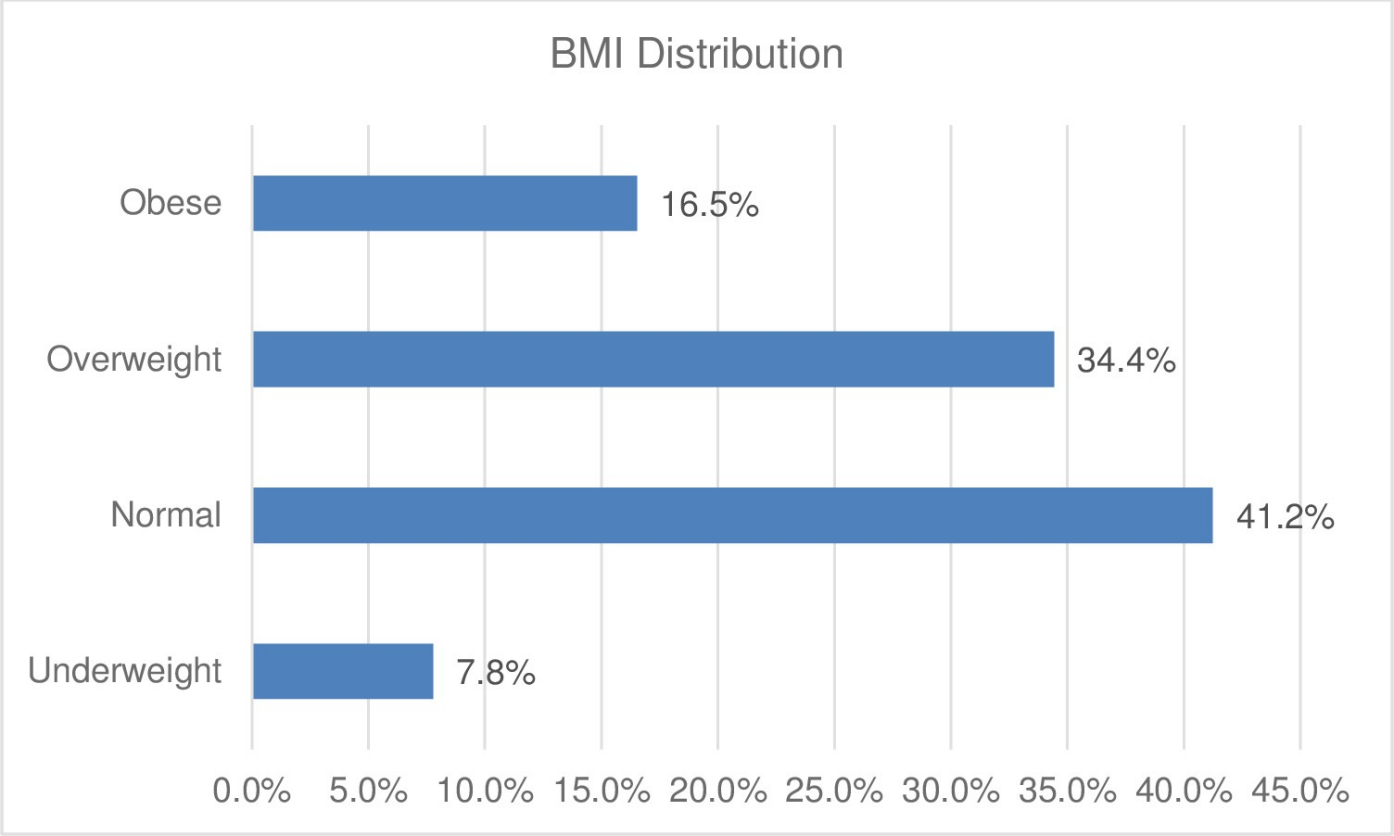
The BMI distribution of the SARHC patients. Underweight: BMI ≤ 18.5; Normal: 18.5 >BMI ≤24.9; Overweight: 25 ≥ BMI < 30; Obese: BMI ≥ 30.

The calculated BMI during 514 visits revealed that 50.9% of patient readings were in the overweight or obese ranges (n = 262). See full distribution of BMI below in Fig 2.

Only 1.1% (n = 6) of patients had a fever, while 12% (n = 58) had tachypnea. Heart rate readings reveal that the majority fell (n = 504, 90.8%) in the normal range (60–100 beats per minute), while 2% were bradycardic (n = 11), and 7.2% (n = 40) were tachycardic.

**Fig 3:**
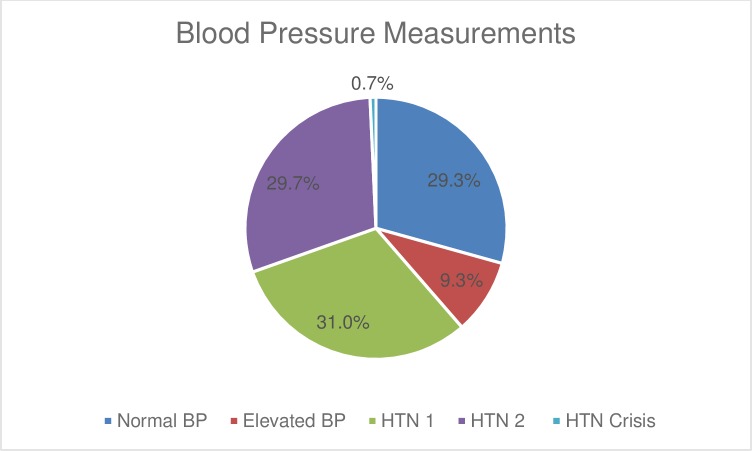
The distribution of blood pressure (BP) readings of the SARHC patients expressed in % of total measurements. Normal BP was a systolic blood pressure (SBP) < 120 mmHg and a diastolic blood pressure (DBP) < 80 mmHg; elevated BP was SBP 120–129 mmHg and DBP < 80 mmHg; Hypertension 1 (HTN 1) was SBP 130–139 mmHg or DBP 80–89 mmHg; Hypertension 2 (HTN 2) was SBP > 140 mmHg or DBP > 90 mmHg, and Hypertensive Crisis (HTN Crisis) was SBP > 180 mmHg or DBP > 120 mmHg.

Regarding BP measurements, 61.4% (n = 337) of the readings were in the hypertensive range, while 9.3% (n = 51) constituted elevated BP, according to the 2017 ACC/AHA guidelines [16] (Fig 3).

In patients whose blood glucose was randomly measured, 23% (n = 20) had a blood glucose level > 200 mg/dL, while 13.8% (n = 12) ranged between 140 and 200 mg/dL, and 63.2% having a blood glucose level < 140 mg/dL.

### Chief complaints (CC)

The total number of CC recorded was 850, indicating that, on average, one patient had 1.9 CC (850 CC/448 patients) during a single visit. Abdominal pain (n = 56) was the most common CC on the list, followed by headache (n = 55), cough (n = 52), medication refill (46), and rash (n = 37). When stratified into broader categories, gastrointestinal complaints (n = 97; 11.4%) were the most common, followed by musculoskeletal complaints (n = 90; 10.6%), upper respiratory symptoms (n = 89, 10.5%), dermatological complaints (n = 74; 8.7%), and constitutional symptoms (n = 55; 6.5%) (Table 3).

**Table 3:** The distribution of chief complaints of the patients at the SARHC.

Chief complaint category	Number (%)
Gastrointestinal complaints	97 (11.4)
Musculoskeletal pain	90 (10.6)
Upper respiratory disease symptoms	89 (10.5)
Dermatological complaints	74 (8.7)
Constitutional symptoms, headache	55 (6.5) each
Dental complaints	54 (6.4)
Follow-up, cardiovascular concerns	48 (5.6) each
Medication refill	46 (5.4)
Genitourinary and obstetric complaints	44 (5.2)
Back pain	37 (4.4)
Other	35 (4.1)
Neurological complaints	28 (3.3)
Vision problems	24 (2.8)
General check-up	20 (2.4)
Psychiatric problems	6 (0.7)
Total	850 (100)

### Diagnoses

The total number of recorded diagnoses for all patients was 586, indicating that, on average, each patient received 1.3 running diagnoses (586 diagnoses/448 patients). Allergic rhinitis (n = 44), viral upper respiratory infections (URI; n = 36), and elevated blood pressure (EBP, n = 32) were the most common running diagnoses, followed by musculoskeletal pain (MSK pain; n = 29) and gastroesophageal reflex disease (GERD, n = 26) (Table 4).

**Table 4:** The top 10 running diagnoses at the SARHC.

Diagnosis	Frequency (%)
Allergic Rhinitis	44 (7.5)
Viral URI	36 (6.1)
EBP	32 (5.5)
MSK pain	29 (4.9)
GERD	26 (4.4)
Arthritis, primary headache	22 (3.8) each
Diabetes mellitus, UTI*	20 (3.4) each
Unknown	17 (2.9)

*UTI: Urinary Tract Infections.

Interestingly, the running diagnoses list differs by country of origin (Table 5).

**Table 5:** List of running diagnoses among the 5 most represented countries.

Country	1^st^ Diagnosis (n)	2^nd^ Diagnosis (n)	3^rd^ Diagnosis (n)
Bhutan	Allergic rhinitis (19)	Headache(14)	Musculoskeletal pain(13)
Iraq	EBP(6)	Allergic rhinitis (4)	Dental caries; Diabetes mellitus (3)
Burma	Viral URI (10)	Musculoskeletal pain(6)	GERD (5)
Iran	Psychiatric issues (5)	Dental infections; GERD; EBP (2) each	
Thailand	Dental infections (4)	Allergic rhinitis (3)	EBP; Rotator Cuff Tendinopathy; Vision problems (2) each

### Dispensed and prescribed medications

**Fig 4:**
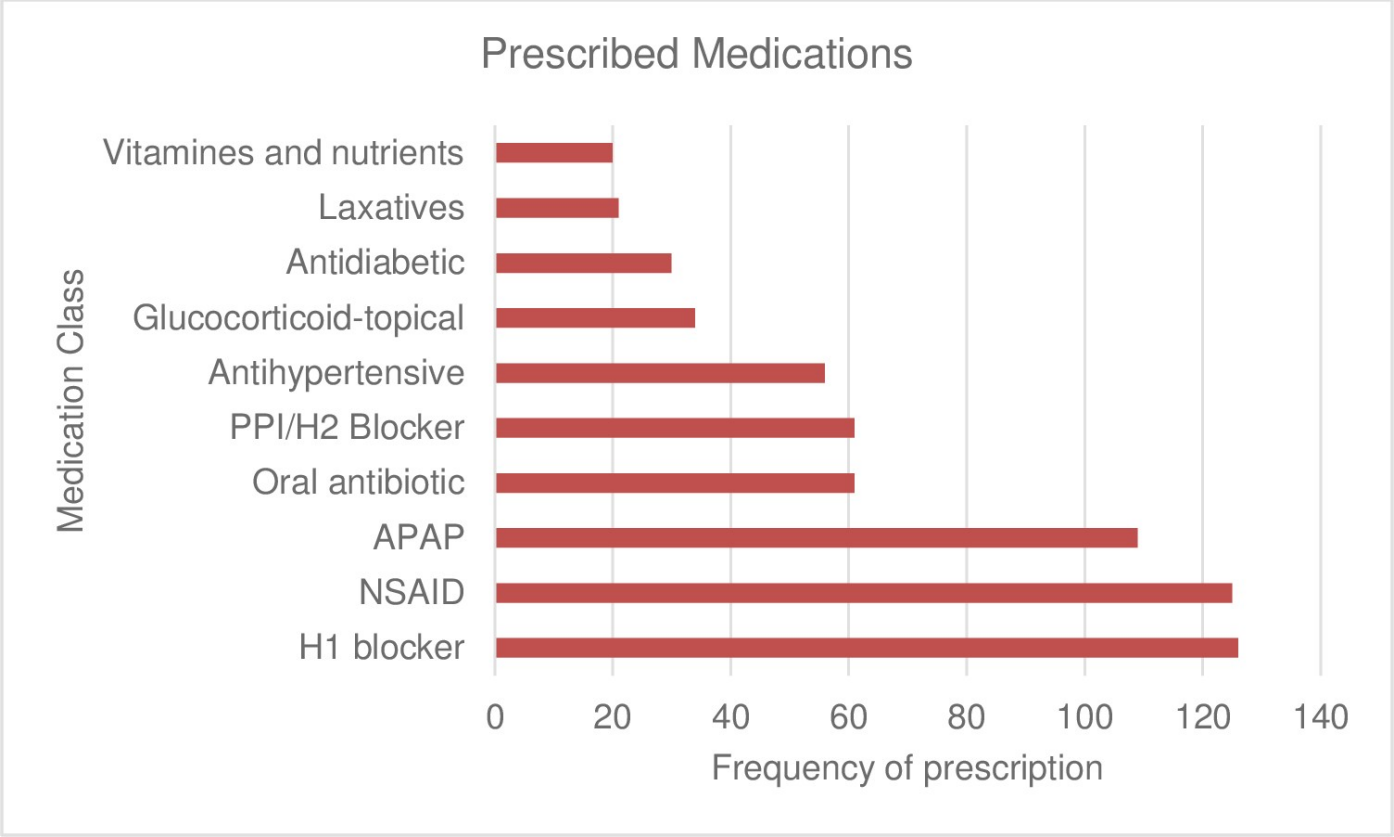
The most commonly prescribed medications at the SARHC. PPI/H2 Blocker: proton-pump inhibitor/Histamine receptor-2 blocker; APAP: acetaminophen; NSAID: non-steroidal anti-inflammatory; H1 blocker: Histamine receptor-1 blocker.

The most commonly dispensed medications correspond to the most common running diagnoses and the most common chief complaints. Interestingly, even though MSK pain and arthritis were very common, our records indicate that only 1 patient was prescribed an opioid-containing analgesic, and 1 patient was given Tramadol (Fig 4). Additionally, the most commonly ordered labs were inspected (S1 Table).

### Tobacco and alcohol use

Forty percent of patients (n = 179) were asked about their tobacco use, and 7.1% (n = 32) said yes. In terms of alcohol use, the question was asked to 38.8% (n = 174) of patients, and only 2.9% (n = 13) reported consuming alcohol (Table 6).

**Table 6:** Tobacco and alcohol consumption of the SARHC patients.

	Tobacco	Alcohol
**No**	32.8%	35.9%
**Yes**	7.1%	2.9%
**Unknown**	60.0%	61.2%

## Discussion

Through our inspection of 731 patient charts at the SARHC, we were able to identify the patients’ demographics, need for interpretation, symptomatology, BMI distribution, vital signs, diagnoses, prescribed medications, and their social histories. Most importantly, our review has revealed that the demographics of the San Antonio refugees are unique when compared to the overall Texas refugee population. This indicates that unique pockets of patients tend to form inside a state. Therefore, applying statewide refugee statistics to citywide populations may be suboptimal. Active patient characterization at individual healthcare centers may be vital for optimal service provision to this extremely diverse population. It is noteworthy to mention that the patients that we usually saw at the SARHC had probably been in the US for at least 6–8 months. This is because during the first 6–8 months after arrival, Medicaid is available to them in Texas. With Medicaid coverage, patients tend to afford going to non-free clinics. However, afterwards, some of them become uninsured, and it is mostly during that time that do we see them at the student-run free clinic.

The age distribution was slightly different from that of the Texas refugees admitted in 2014[4]. In the latter group, people ages 1–10 were the most represented segment, while 41-50-year-old patients constituted the majority of our sample. One explanation for why children are not as represented in our sample may be that CHIP or Medicaid is available to them and may allow them to see a regular primary care provider, thus not necessitating visits to the SARHC. Further investigation into this phenomenon may yield interesting observations in the future.

While the ethnically-Nepali Bhutanese refugees constituted the majority of the SARHC patients (n = 107, 43.1%), they only represented 7% of the sample of Texas refugees [4]. This could be because most immigrant communities tend to live together, and perhaps many of the Texas Bhutanese relocate to Bexar County. Another potential factor is that many of the Bhutanese refugees live within walking distance off the clinic, thus leading to a relative over-representation of this subpopulation.

In addition to the aforementioned population, Iraqi (n = 35, 14.1%) and Burmese (n = 30, 12.1%) patients were well represented in both samples, where they comprise the majority of the Texas refugees [4]. Consequently, the first language distribution in our sample followed that of the countries of origin, with Nepali, Arabic and Burmese being the most common, respectively. Interestingly, even though 19 patients came from Iran, only 16 spoke Farsi as their 1st language. This could be because they were mostly of the Mandaean minority and they hailed from Iraq-bordering areas.

Interpretation was needed by 49.8% of patients and preferred by 5.5%, while only 44.8% responded by saying “No-I do not need an interpreter.” The latter figure is surprisingly low, especially when compared to the rest of the incoming refugees to Texas [4], where almost 90% needed interpretation. One reason is that some of the patients who did not need an interpreter may have actually been accompanied by an English-speaking relative or friend. Another plausible explanation is that some of the student volunteers were multi-lingual, which may have omitted the need for an interpreter. Although less commonly, some patients had probably been in the US long enough to have learned conversational English. Yet, the fact that more than 50% of patients needed an interpreter and that they come from 26 countries point to the dire need for a reliable phone or online interpretation service at such a clinic. More importantly, it would be interesting to see how this need compares to other refugee-serving healthcare facilities.

Alarmingly, 86.6% of the SARHC patients were uninsured. According to the US Census Bureau, the percentage of the uninsured US population is 9.1% and 8.8% in 2015 and 2016, respectively [17]. The fact that the uninsured rate is significantly higher among the SARHC patients may be due to selection bias since patients who have insurance may not use the SARHC as a safety net.

Other factors contributing to the high rate of uninsured may include lack of linguistic proficiency to enroll in a health insurance plan, inadequate understanding of the US healthcare system, and limited means [14].

In terms of BMI, 50.9% (n = 262) of the SARHC patients were either overweight or obese, compared to 70.2% of US adults [18]. This is consistent with another Bhutanese population study [19] and the Iraqi refugees’ healthcare profile [20]. Additionally, the prevalence of obesity in our sample was 16.5%, which is lower than that of the general US population at 37.7% [18]. However, the fact that more than half of our patients were overweight or obese is alarming. This observation also shows that non-communicable chronic diseases are prevalent among refugees and that closer attention needs to be paid to address these issues. Furthermore, this phenomenon may be linked to the fact that most of the SARHC patients had been in the US for at least 6–8 months after arrival, as mentioned above. Perhaps educating refugees early in the resettlement process about healthy diet and the importance of exercise may be warranted. For our clinic, distributing educational pamphlets in various languages is feasible or having group presentations on making nutritious food choices and the importance of regular exercise. Having a nutrition consultant for this unique population may also be beneficial in light of the diverse diets and customs of the patients.

Most interesting were the BP readings, 61.4% of which fell in the hypertensive range, and EBP was the third most common running diagnosis in our patient population. This figure seems to be higher than what is reported in other studies [21]. One reason is the fact that we used the 2017 ACC/AHA guidelines, which increased the sensitivity of abnormal BP measurements [16]. Other factors potentially include white coat hypertension and anxiety from being in an unfamiliar clinical setting. Regardless, the high prevalence of abnormal readings is concerning and points to the possibility that US healthcare providers may be overlooking and, therefore, undertreating chronic noninfectious diseases among refugees.

Repeating BP measurements, encouraging follow-up for such patients, and providing patient education about the importance of diet and exercise in controlling BP are feasible measures that can be implemented and have been proven to be effective in other settings [22].

Furthermore, 36.8% of patients had a blood glucose level > 140 mg/dL. While this was a random glucose reading and not after fasting, it remains a bit concerning. According to the American Diabetes Association, a blood glucose > 200 after two hours of having eaten may indicate diabetes mellitus, meaning that at least 23% of patients may have diabetes and 13.8% may have impaired glucose tolerance [23]. This is consistent with other studies [22, 24] reporting that diabetes mellitus is a highly prevalent condition among refugees.

On average, each patient at the SARHC had almost 2 chief complaints per visit, with abdominal pain, headache, cough, medication refill, and rash being the most common, respectively. In terms of diagnoses, allergic rhinitis, viral URI, EBP, MSK pain, and GERD were the most common, respectively. However, the overlapping of symptoms between allergic rhinitis and viral URI could affect the accuracy of reporting [25]. Disease distribution differed among countries of origin. However, elevated blood pressure, allergic rhinitis and dental complaints seemed to be the most frequent problems across the five most represented countries.

While abdominal pain was the most common CC, GERD was the 5th most common diagnosis. One explanation for this discrepancy may be because abdominal pain can be a symptom of other conditions, thus not getting a distinct designation as a single diagnosis. It may be prudent to further investigate this phenomenon. Another point to consider is that San Antonio is number 23 on the list of Worst Cities for Allergies in the US [26]. Texas has an estimated 15.5–38.2%, prevalence of allergic rhinitis, one of the highest in the nation [27].Further, studies have shown that there is an increased prevalence of allergic rhinitis in developing countries [28–30]. Both observations could explain why allergic rhinitis is the most common diagnosis in the SARHC.

Most importantly, the analysis of the diagnoses revealed a higher burden of non-communicable diseases in this population, which seems to be consistent with other studies [19, 21, 31], yet at odds with others [4]. The fact that a significant portion of the patients had elevated BP readings hints at the potentially high prevalence of hypertension in this population. Additionally, one reason infectious diseases were not very prevalent may be due to the rigorous screening process refugees undergo and the anti-helminthic prophylactic treatment they receive prior to coming to the US [20, 32, 33]. Another important point to consider is that despite the relatively high percentage of patients with high glucose readings, diabetes mellitus was only an established diagnosis in 3.4% (n = 20 patients). This observation may indicate the need for an active approach in screening for diabetes among refugees overall, especially at the clinic.

The ethnically Nepali Bhutanese population’s most common diseases were allergic rhinitis, headaches, and MSK pain. Interestingly, the prevalence of non-communicable diseases seemed to be different from that at the Grady Refugee Clinic in Atlanta, GA [19]. However, the results were consistent with the 2014 US Department of Health and Human Services report [33], with URI being the most common infectious disease, and EBP being the most common non-communicable disease.

Among the Iraqi refugees, EBP was the most common diagnosis, followed by allergic rhinitis, diabetes mellitus, and dental caries. This is consistent with a report by the US Department of Health and Human Services (HHS), indicating that 33% had hypertension [20] at a single screening in Jordan, and 44% were pre-hypertensive. This also means that healthcare providers should have a higher index of suspicion for hypertension and diabetes mellitus in this population.

Viral URI, MSK pain, and GERD were the top 3 diagnoses in the Burmese population. According to HHS, Texas houses the largest population of Burmese refugees (16,689, 14.2%) in the United States. Viral URI (n = 10) and tinea pedis (n = 3) were the most common communicable diseases. GERD, Allergic rhinitis, and dental problems were among the most common non-communicable diseases, consistent with HHS reporting dental issues as the most common. According to the HHS, Hepatitis B and intestinal parasites are a significant burden in this population. Yet, none of our patients were diagnosed with either disease. This could possibly be due to refugees receiving prophylactic anti-helminthic treatment prior to coming to the US. Another possibility is underdiagnosis. Therefore, active case seeking may be necessary in this population for parasites and Hepatitis B [32].

The Iranian population had the highest burden of psychiatric diseases (depression n = 2, insomnia n = 2, anxiety n = 1), followed by EBP, dental concerns, and GERD (n = 2 for each). In a study done in California on Iranian refugees, they seemed to have a higher burden of diabetes mellitus and tobacco use than the general California population [34]. While the results may not be congruent, psychiatric diseases and tobacco use have been linked [35, 36]. Therefore, a screening mechanism for psychiatric conditions, such as PHQ-9, may need to be adopted when evaluating this population of refugees. Another important implication is understanding effective treatment strategies for psychiatric conditions in this population.

Thai refugees had dental infections (n = 4), allergic rhinitis (n = 3), and EBP, rotator cuff tendinopathy, and vision problems (n = 2 for all) as the most common problems.

Interestingly, mental health conditions were not highly prevalent in our refugee population despite the many reports indicating their prevalence among refugees, especially the ones displaced by wars [37–41]. Post-Traumatic Stress Disorder (PTSD) [38, 39], anxiety, depression [40], and somatization [41] were among the common conditions afflicting refugees. In a meta-analysis by Bogic et al, the prevalence of depression ranged between 2.3–80%; PTSD was found in 4.4–86%, and anxiety disorder fell in the 20.3–88% range [37]. One explanation for the low prevalence of such diseases in our cohort may be due to underdiagnosis, which is a well-known phenomenon [37–38, 41]. In fact, one potential explanation for the high incidence of musculoskeletal complaints, headaches, and constitutional symptoms in our cohort’s list of chief complaints is somatization. Somatization, with a prevalence of 33% among primary care visits of non-migrants and an even higher prevalence among refugees, has been shown to manifest as the aforementioned chief complaints [41]. Consequently, somatization may also account for having MSK pain, arthritis, headaches, and an “unknown diagnosis” among the most common diagnoses at our clinic. Thus, it is prudent for healthcare providers everywhere to have a high index of suspicion of mental health diseases, especially somatization, when providing care to refugees, which can result in better patient outcomes [40].

The list of the most commonly prescribed medications followed the trend of the most common diagnoses. Given that allergic rhinitis and viral URI were the most common diseases, H1 blockers were the most common medications (n = 126), followed by NSAIDs (125), and APAP (n = 109). APAP and NSAIDs were also given to address MSK pain and arthritis. Interestingly, oral antibiotics were the 4th most commonly prescribed medication (n = 61) even though UTI (n = 20) was 9^th^ on the list of common diagnoses and was the most common infectious disease on the list following viral URI. This may be due to over-prescription of antibiotics even for viral URI or allergic rhinitis patients. This also means that healthcare providers at the clinic and in general should be more cautious when prescribing antibiotics.

PPIs and H2 blockers were next, consistent with GERD being the 5^th^ diagnosis on the list. Antihypertensive medications correspond to EBP being third on the list. Interestingly, despite having MSK pain high on the list, only one opioid-containing analgesic was given, and tramadol was prescribed to one other patient, possibly indicating that the SARHC providers were mostly utilizing non-narcotic analgesics, such as APAP and NSAID, for pain control. It may also be because refugees are not aware of or are more wary of narcotics. It would be interesting to investigate this phenomenon at other refugee-serving centers. This review did not include non-pharmacologic treatments that may have been recommended for the MSK conditions, such as physical therapy.

Even though GERD is common, only 9 patients were tested for *H*. *pylori*, even though the burden of such infections is higher in developing countries [42]. Further, socioeconomic status and living conditions, such as overcrowding, sharing a bed, and lack of running water, are all conditions that some of the refugee populations are exposed to, which have been linked to higher incidence of *H*. *pylori* infection [43, 44]. The exact reason is unknown as to why not many other patients had been tested for it or had a recorded test result. One contributing factor is the limited resources of the clinic given that it is a student-run faculty-supervised, free clinic. However, this point was included as part of the post-study, practice-changing recommendations. It may also be used to alert other immigrant-heavy healthcare practices in order to pay more attention to testing for *H*. *pylori*.

Another interesting finding is the lack of overt nutritional deficiencies diagnoses. One explanation is that the SARHC patients may have already been in the USA long enough to have corrected those deficiencies. Another possibility is that some patients were treated empirically with multivitamin supplements, likely resulting in underreporting of the nutrient deficiencies.

Social history responses were not recorded for 60% of patients. This is concerning since refugees can have a rate of hazardous drinking comparable to that in the Western hemisphere [45]. This population also has a prevalence of tobacco use of 20% among men, with 12% of respondents having smoked within the last month [46]. The fact that only 40% of the SARHC patients had been asked about their social history indicates the need to do it more consistently. Also, one reason alcohol use may be underreported is because of cultural stigmatization among certain refugee communities [47]. Therefore, healthcare providers should be encouraged to utilize careful phrasing when asking these questions rather than not asking them at all.

To the best of our knowledge, our study is the first one to evaluate the demographics and health profiles and needs of refugees in an outpatient student-faculty collaborative practice, as studies often seem to focus on the emergency departments [10] or refugee camps [22]. Further, our study focuses on the common complaints among the five most represented groups. Additionally, instead of simply focusing on infectious diseases among refugees [7, 8], we evaluated infectious and noninfectious conditions. Our study was also unique in that it inspected the vital signs of patients and their treatments, important aspects that seem to be overlooked in other studies. Our findings helped us devise practice recommendations to the SARHC protocol, the effectiveness of which will be assessed upon future analysis of the SARHC patients after recommendations implementation. Ultimately, our data could help prepare and alert other refugee healthcare providers as to what issues they might face and how they could provide informed, targeted, and quality care for refugees.

## Limitations

This study focuses only on the San Antonio refugees who are also patients at the SARHC, thus limiting the generalizability of our findings. Furthermore, some demographic data, especially country of origin, language, and insurance status, were missing from some patients’ charts. This is because the electronic medical record at the SARHC used until October 2015 did not explicitly include fields to collect such information. Another limitation pertaining to the most common diagnoses is the small sample size of all of the non-Bhutanese subpopulations, which may affect the applicability of some findings.

Furthermore, it was not possible to assess the refugees’ disease burden upon arrival to the US, and compare that to what they developed while being in the US. This is because our clinic is a free resource to some of the refugees after they have settled in the US. In Texas, refugees are covered under Medicaid for the 1st 6–8 months, after which they lose their coverage. It is usually after that coverage expiration that we see some of those patients at the SARHC. At that point, it is hard to assess the pre-resettlement disease burden accurately.

Additionally, it was not feasible to determine whether those refugees resettled initially to San Antonio. That was because the SARHC is a student-run free clinic that is not affiliated with an official state or local resettlement agency that could have provided such information. Additionally, refugees are usually free to move among states once they resettle in the US.

## Supporting information

S1 Table Ordered labs and other studies. (DOCX)
