# Supplementary material for: San Antonio refugees: Their demographics, healthcare profiles, and how to better serve them
Source: PLoS One. 2019 Feb 19;14(2):e0211930. doi: 10.1371/journal.pone.0211930 (PMC6380579; doi:10.1371/journal.pone.0211930)
Supplement: S1 Table — (DOCX) [file pone.0211930.s001.docx]

| **Ordered Lab** | **Frequency** |
| --- | --- |
| Finger stick glucose | 85 |
| Urinalysis | 63 |
| Complete Blood Count | 53 |
| Complete Metabolic Panel | 50 |
| Lipid panel | 29 |
| Hemoglobin A1c | 23 |
| Thyroid Stimulating Hormone | 20 |
| Basic Metabolic Panel | 14 |
| Urine BhCG | 13 |
| *Helicobacter pylori* antigen | 9 |
